# Circulating serum metabolites as biomarkers and predictors of residual feed intake in lactating dairy cows

**DOI:** 10.1038/s41598-025-85610-1

**Published:** 2025-01-11

**Authors:** Dagnachew Hailemariam, Ghader Manafiazar, Christine Baes, Flavio Schenkel, Filippo Miglior, Paul Stothard, Graham Plastow

**Affiliations:** 1 Department of Agricultural, Food and Nutritional Science, University of Alberta, Edmonton, AB T6G2P5 Canada; 2 Department of Animal Science and Aquaculture, Faculty of Agriculture, Dalhousie University, Halifax, NS Canada; 3 Centre for Genetic Improvement of Livestock, University of Guelph, Guelph, ON Canada

**Keywords:** Serum metabolome, Residual feed intake, Biomarkers, Prediction, Metabolomics, Metabolism

## Abstract

This study explored the potential of circulatory serum metabolite profiles to increase understanding of the physiology of feed efficiency and identify biomarkers to predict residual feed intake (RFI) in lactating Holsteins. Serum metabolite profiles were compared in high (*n* = 20) and low RFI (*n* = 20) cows at early, mid, and late lactation stages. The low RFI cows had decreased (*P* < 0.05) concentrations of dodecanoylcarnitine, dodecenoylcarnitine, dodecanedioylcarnitine, tetradecanoylcarnitine, succinic acid, trimethylamine N-oxide, betaine, and increased concentrations of p-Hydroxyhippuric acid, hydroxysphingomyeline C16:1, phosphatidylcholine diacyl C40:6, and glutarylcarnitine at early lactation. A similar comparison at mid lactation stage showed altered serum concentrations of 26 metabolites that fall into the categories of acyl carnitines, glycerophospholipids, biogenic amines, amino acids, and organic acids. At late lactation, fewer sets of metabolites were significantly affected by RFI grouping. Receiver operator curve analyses identified p-Hydroxyhippuric acid as the top biomarker at early lactation and acetylornithine at mid and late lactation. Models based on sets of serum metabolites in early, mid, and late lactation stages predicted RFI with a validation coefficient of determination of 0.54, 0.68, and 0.64, respectively. This study demonstrated the potential of circulatory serum metabolites as biomarkers and predictors of RFI in lactating dairy cows.

## Introduction

Residual feed intake (RFI) is a measure of feed efficiency and is defined as the difference between actual and predicted feed intake after accounting for production and maintenance^1^. Low RFI cows are the most feed efficient and high RFI cows are the least efficient. RFI is a composite trait and is defined to be independent of body size, growth rate, body composition measures, and milk yield. Biological mechanisms documented to be associated with RFI include feeding behavior, digestion and methane production, rumen microbiome structure and functioning, energy metabolism, protein turnover, hormone regulation, and body composition^2–5^. However, feeding and digestive-related mechanisms could be associated with RFI mainly because they co-vary with feed intake. The role of these mechanisms as true determinants of animal variability in feed efficiency could be minor^5^.

Concentrations of circulating serum metabolites have been utilized to develop biomarkers of diseases^6,7^, milk production and quality^8^, and heat stress^9^ in dairy cattle. Recently, serum biomarkers of RFI in beef cattle with high sensitivity and specificity were identified to distinguish high and low RFI cows^10^. In addition, promising work has been done on metabolomics in beef cattle which showed that energy and protein metabolism, as well as metabolism of urea and methane, were associated with RFI^11^. A serum-based metabolic signature associated with feed efficiency in beef cattle before entering the feedlot has also been reported^12^. However, the potential of the serum metabolome to reveal the physiological differences between divergent RFI groups, to identify biomarkers of RFI or set of metabolites that can be used to predict individual RFI phenotypes in lactating dairy cows are not yet explored.

RFI estimation is costly and difficult and this limits the application of RFI to improve feed efficiency. Given RFI is a hard-to-measure trait, the discovery of cheap and rapid tools that can assist the ranking of individuals based on feed efficiency or directly predicting individual RFI phenotypes could serve as a key alternative. Prediction of RFI from descriptive, performance, sensor-derived behavioral, and some blood analytes (glucose, BHB, triglycerides, and nonesterified fatty acids) resulted in poor prediction performance of the models in dairy cattle^13^. In our previous study, we identified candidate biomarkers and developed models that can be used to predict RFI using milk metabolome^14^. Here, we report serum-based biomarkers and prediction models of RFI that could provide alternative RFI testing tools. Blood sampling provides systemic insights that may not be obtainable from milk, offering an alternative or complementary approach. The objective of the study was to elucidate physiological differences, identify biomarkers, and develop prediction models of RFI at early, mid, and late lactation using targeted serum metabolome.

## Results

### Multivariate analysis on longitudinal serum metabolome profiles

**Fig. 1:**
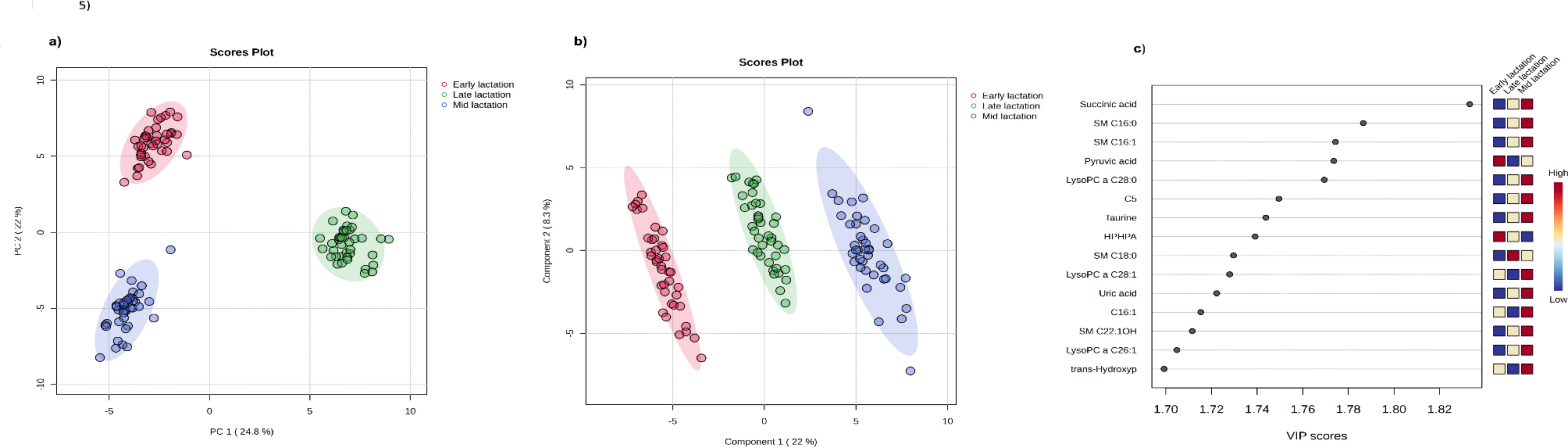
Multivariate analyses (PCA, PLS-DA and VIP) on serum metabolome profiles collected from lactating Holsteins (*n* = 40) at early (50.6 ± 4.6), mid (150.8 ± 2.6) and late (240.4 ± 3.8) lactation stages. The PCA (**a**) and PLS-DA (**b**) analyses shows clear separation between the lactation stages and the VIP score shows the top 15 metabolites that distinguish the lactation stages (**c**).

Multivariate analyses on serum metabolite profiles (from a total of 120 samples) at early, mid and late lactation stages of Holstein dairy cows (*n* = 40) were undertaken to examine the impact of lactation on serum metabolite profiles (Fig. 1). The principal component analysis on serum metabolite profiles showed a clear separation between the lactation stages (Fig. 1a). The PLS-DA also showed distinct grouping of cows based on the lactation stage specific metabolite profiles (Fig. 1b). The top 5 metabolites that distinguished the three stages of lactation include, succinic acid, SM C16:0, SMC16:1, pyruvic acid and lysoPC a 28:0 with VIP > 1.7 (Fig. 1c).

### Changes in metabolite profiles between RFI groups at early lactation

The list of serum metabolites significantly (*P* < 0.05) affected by RFI grouping at early (50.6 ± 4.6 DIM) lactation stage are presented in Table 1. Comparison of high and low RFI groups at the early lactation stage revealed that medium-chain acylcarnitines were significantly elevated in high RFI cows. These include, dodecanedioylcarnitine (C12-DC), dodecenoylcarnitine (C12:1), dodecanoylcarnitine (C12) and tetradecanoylcarnitine (C14). Conversely, the concentration of glutarylcarnitine (C5-DC) was significantly decreased in high RFI cows. Among the organic acids, succinic acid was increased in high RFI cows, whereas, p-Hydroxyhippuric acid decreased in high RFI cows as compared to the low RFI. The high RFI cows had increased (*P* < 0.05) betaine and trimethylamine N-oxide serum concentrations compared to the low RFI cows. A similar comparison for hydroxysphingomyeline C16:1 (SM(OH) C16:1), and phosphatidylcholine diacyl C40:6 (PC aa C40:6) revealed that high RFI cows had decreased concentrations of these metabolites. Methylmalonic acid, phosphatidylcholinediacyl C36:6 (PC aa C36:6), serotonin, hydroxytetradecadienylcarnitine (C14:2-OH), hydroxypropionylcarnitine (C3-OH), valerylcarnitine (C5), lysophosphatidylcholineacyl C16:1 (lyso PC C16:1), octanoylcarnitine (C8), nonaylcarnitine (C9), and tiglylcarnitine C5:1 were the metabolites that tended to differ between high vs. low comparison (Table 1).

**Table 1:** Least square means (LSM) and standard errors (SE) for the serum metabolite concentrations that were significantly different and tended to differ between high (*n* = 20) and low (*n* = 20) RFI cows at early (50.6 ± 4.6 DIM) lactation stage of Holstein cows.

Metabolite ( µM)	High RFI		Low RFI		*P*-value ^a^	Fixed effects^b^
	LSM	SE	LSM	SE		
p-Hydroxyhippuric acid	0.0732	0.0043	0.0989	0.0043	1.62E-04	
Tetradecanoylcarnitine (C14)	0.0066	0.0006	0.0045	0.0004	2.93E-03	
PC aa C40:6	1.15	0.119	1.71	0.163	3.91E-03	MY, P
Dodecanoylcarnitine (C12)	0.0396	0.0017	0.0332	0.0014	5.55E-03	AFC
Dodecenoylcarnitine (C12:1)	0.112	0.0078	0.081	0.0078	8.99E-03	
Dodecanedioylcarnitine (C12-DC)	0.0143	0.001	0.0108	0.0012	1.47E-02	
Succinic acid	2.31	0.125	1.87	0.163	1.95E-02	MY, P
SM (OH) C16:1	13.2	0.896	16	0.928	3.56E-02	MY
Betaine	30.5	3.84	20.8	3.42	4.07E-02	MY
Glutarylcarnitine (C5 –DC)	0.0201	0.0013	0.0241	0.0018	4.13E-02	P, AFC
Trimethylamine N-oxide	83	10.8	51.9	12.2	4.91E-02	HS
Methylmalonic acid	0.515	0.0492	0.401	0.0404	5.02E-02	MY
PC aa C36:6	0.762	0.0697	0.948	0.0681	5.60E-02	MY
Serotonin	1.79	0.526	4.01	1.425	5.84E-02	AFC
Hydroxytetradecadienylcarnitine (C14:2-OH)	0.0039	0.0003	0.0047	0.0003	6.43E-02	AFC
Hydroxypropionylcarnitine (C3-OH)	0.0539	0.0019	0.0489	0.0021	6.50E-02	
Valerylcarnitine (C5)	0.0123	0.0008	0.0142	0.0009	7.01E-02	P
LysoPC a C16:1	6.57	0.572	8.09	0.593	7.18E-02	AFC
Octanoylcarnitine (C8)	0.0154	0.0012	0.0127	0.001	9.01E-02	
Nonaylcarnitine (C9)	0.0124	0.001	0.0151	0.0012	9.25E-02	
Tiglylcarnitine (C5:1)	0.0243	0.0009	0.022	0.0009	9.77E-02	

^a^ significantly different: P-value < 0.05, tendency: 0.05 < *P* < 0.1, ^b^ Fixed effects that were significant (*P* < 0.05), MY = month and year of sampling, P = parity, AFC = age at first calving, HS = health status.

### Changes in metabolite profiles between RFI groups at mid lactation

Similarly, the high and low RFI groups were compared at the mid (150.8 ± 2.6 DIM) lactation stage and the concentrations of 26 serum metabolites were significantly (*P* < 0.05) different between the RFI groups (Table 2). Moreover, 6 metabolites tended to differ between the groups. Among the 26 metabolites, 13 metabolites were increased and the other 13 were decreased in the high RFI compared to the low RFI groups. Acetylornithine, alpha-aminoadipic acid, p-Hydroxyhippuric acid, sarcosine, betaine, sphingomyeline C20:2 (SM 20:2), methylglutarylcarnitine (C5-M-DC), sphingomyeline C18:1 (SM C18:1), nonaylcarnitine (C9), tetradecenoylcarnitine (C14:1), sphingomyeline C16:1 (SM C16:1), hydroxysphingomyeline C16:1 (SM(OH)C16:1), sphingomyeline C18:1 (SM C18:1) were increased and octanoylcarnitine (C8), hexanoylcarnitine (C6), tetradecanoylcarnitine (C14), octadecanoylcarnitine (C18), dodecanoylcarnitine (C12), hydroxyoctadecenoylcarnitine (C18:1OH), hydroxyhexadecenoylcarnitine (C16:1OH), lysoPhosphatidylcholine acyl C28:0 (lysoPC a C28:0), lysoPhosphatidylcholine acyl C14:0 (lyso PC a C14:0), sphingomyeline C18:0 (SM C18:0), methionine-sulfoxide, phosphatidylcholine diacyl C40:6 (PC aa C40:6) and trimethylamine N-oxide were decreased in high RFI compared to the low RFI cows. Sphingomyeline C16:0 (SM C16:0), citrulline, hydroxysphingomyeline C14:1(SM(OH) C14:1), homovanillic acid, phosphatidylcholine diacyl C38:0 (PC aa C38:0) and propionylcarnitine (C3) tended to differ between high and low RFI cows.

**Table 2:** Least square means (LSM) and standard errors (SE) for the serum metabolite concentrations that were significantly different and tended to differ between high (*n* = 20) and low (*n* = 20) RFI groups at mid (150.8 ± 2.6 DIM) lactation stage of Holstein cows.

Metabolite (µM)	High RFI		Low RFI		*P*-value^a^	Fixed effects ^b^
	LSM	SE	LSM	SE		
Acetylornithine	5.88	0.266	3.7	0.266	2.56E−08	
Alpha-Aminoadipic acid	3.71	0.346	2.16	0.161	1.49E−04	MY
SM C18:1	24.6	1.1	18.4	1.1	3.15E−04	
Octanoylcarnitine (C8)	0.0125	0.0016	0.0225	0.0028	1.14E−03	
LysoPC a C28:0	1.51	0.0812	1.19	0.0812	1.68E−03	
Methylhistidine	8.53	0.452	10.61	0.461	2.89E−03	MY
SM C20:2	2.27	0.119	1.73	0.119	3.10E−03	
Hexanoylcarnitine (C6)	0.0186	0.0026	0.0347	0.0049	3.62E−03	
Methionine-Sulfoxide	1.9	0.296	3.64	0.568	5.51E−03	
PC aa C40:6	0.985	0.121	1.422	0.141	1.02E−02	P
p-Hydroxyhippuric acid	0.1039	0.0038	0.0895	0.0038	1.06E−02	
Methylglutarylcarnitine (C5-M-DC)	0.0229	0.0013	0.0183	0.0011	1.45E−02	
Tetradecanoylcarnitine (C14)	0.004	0.0005	0.0063	0.0009	1.92E−02	
Octadecanoylcarnitine (C18)	0.0165	0.0025	0.0253	0.0032	2.24E−02	MY, AFC
Octadecenoylcarnitine (C18:1)	0.0097	0.001	0.0136	0.0012	2.48E−02	
Sarcosine	0.6	0.047	0.449	0.0568	2.79E−02	P, AFC
LysoPC a C14:0	8.53	0.452	10.61	0.461	2.82E−02	MY
Nonaylcarnitine (C9)	0.0163	0.0014	0.0124	0.0011	2.86E−02	
Hydroxyhexadecenoylcarnitine (C16:1-OH)	0.0059	0.0006	0.0081	0.0007	3.03E−02	MY
SM C16:1	18.4	0.792	15.8	0.792	3.06E−02	
SM C16:1OH	18	0.736	15.8	0.736	3.49E−02	
Tetradecenoylcarnitine (14:1)	0.0458	0.002	0.04	0.0024	3.80E−02	
Betaine	57.6	8.21	38.2	6.4	3.86E−02	P
Dodecanoylcarnitine (C12)	0.0323	0.0014	0.0366	0.0016	4.68E−02	
Trimethylamine N-oxide	78.8	15.1	123.4	15.1	4.81E−02	AFC
SM C18:0	21.4	0.701	19.4	0.701	4.89E−02	
SM C16:0	165	6.62	147	6.62	6.62E−02	
Citrulline	86	3.84	76.3	4.64	7.89E−02	
SM C14:1OH	16.8	0.761	14.9	0.761	8.71E−02	
Homovanillic acid	0.182	0.0274	0.246	0.0371	9.08E−02	
PC aa 38:0	1.34	0.117	1.59	0.096	9.27E−02	MY
Propionylcarnitine (C3)	0.21	0.0252	0.276	0.0265	9.78E−02	MY, AFC

^a^ significantly different: P-value < 0.05, tendency: 0.05 < *P* < 0.1, ^b^ Fixed effects that were significant (*P* < 0.05), MY = month and year of sampling, P = parity, AFC = age at first calving, HS = health status.

### Changes in metabolite profiles between RFI groups at late lactation

At the late stage (240.4 ± 3.8 DIM) of lactation, 10 metabolites were significantly (*p* < 0.05) different between high and low RFI cows where acetylornithine, citrulline, alpha-aminoadipic acid, betaine, choline, lysoPhosphatidylcholine acyl C28:0 (lysoPC a C28:0), lysoPhosphatidylcholine acyl C28:1 (lysoPC a C28:1), sphingomyeline C18:1 (SM C18:1) were increased, and methylhistidine and fumaric acid were decreased in high RFI as compared to low RFI cows (Table 3). p-Hydroxyhippuric acid, carnosine, succinic acid, dimethylarginine, and putrescine, asymmetric dimethylarginine, valerylcarnitine (C5), tiglylcarnitine (C5:1), sphingomyeline C20:2 (SM C20:2), hexadecanoylcarnitine (C16), phosphatidylcholine diacyl C40:6 (PC aa C40:6) tended to differ between high vs. low RFI groups.

**Table 3:** Least square means (LSM) and standard errors (SE) for the serum metabolite concentrations that are significantly different and tended to differ between high (*n* = 20) and low (*n* = 20) RFI groups at late (240.4 ± 3.8 DIM) lactation stage of Holstein cows.

Metabolite ( µM)	High RFI		Low RFI		*P*-value^a^	Fixed effects ^b^
	LSM	SE	LSM	SE		
Acetylornithine	3.27	0.571	2.04	0.386	1.29E-03	P,
lysoPC a C28:0	1.19	0.137	0.85	0.148	2.25E-03	
Fumaric acid	0.944	0.0838	1.413	0.1253	2.70E-03	
Citrulline	97.9	5.27	80.6	5.84	5.18E-03	
alpha-Aminoadipic acid	2.24	0.389	1.42	0.389	1.17E-02	
Betaine	87.3	11.97	54.7	8.79	1.52E-02	P
lysoPC a C28:1	1.57	0.101	1.29	0.115	2.26E-02	MY, P, AFC
SM C18:1	22.8	2.71	18.3	2.92	3.50E-02	P, HS, MY
Choline	9.01	1.09	7.26	1.18	3.82E-02	
Methylhistidine	9.61	0.475	10.98	0.475	4.80E-02	
PC aa C40:6	1.14	0.101	1.37	0.151	5.30E-02	P
Hexadecadienylcarnitine (C16:2)	0.0067	0.0016	0.0044	0.0017	6.04E-02	
p-Hydroxyhippuric acid	0.0845	0.0042	0.0754	0.0047	6.25E-02	
SM C20:2	2.04	0.285	1.63	0.279	6.53E-02	
Carnosine	17.8	0.852	15.9	0.697	8.39E-02	MY
Succinic acid	1.64	0.0737	1.83	0.0823	8.93E-02	
Valerylcarnitine (C5)	0.0702	0.0047	0.0897	0.0047	9.02E-02	
Tiglylcarnitine (C5:1)	0.022	0.0011	0.0248	0.0011	9.02E-02	
Asymmetric Dimethylarginine	0.567	0.0414	0.467	0.0414	9.54E-02	
Putrescine	0.1078	0.014	0.0915	0.0131	9.83E-02	

^a^ significantly different: P-value < 0.05, tendency: 0.05 < *P* < 0.1, ^b^ Fixed effects that were significant (*P* < 0.05), MY = month and year of sampling, P = parity, AFC = age at first calving, HS = health status.

### Metabolite concentrations affected by RFI grouping across time points

**Fig. 2:**
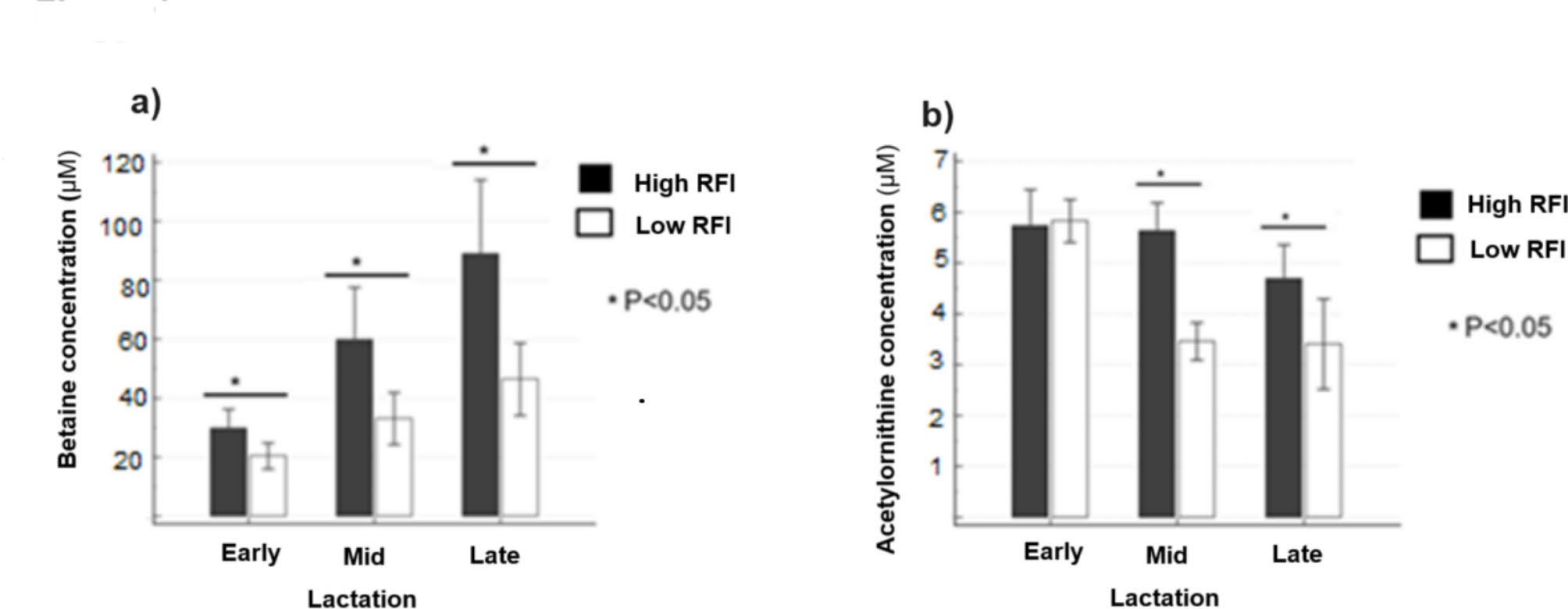
The serum concentration of betaine (**a**) and acetylornithine (**b**) in high and low RFI groups of cows at early (50.6 ± 4.6 DIM), mid (150.8 ± 2.6 DIM) and late (240.4 ± 3.8 DIM) lactation stages.

The serum concentrations of betaine at early, mid, and late lactation were consistently increased (*P* < 0.05) in high RFI cows (Fig. 2a). Betaine also showed an increasing trend in concentration from early to late lactation in both high and low RFI groups. Acetylornithine was not different between RFI groups at early lactation but significantly (*P* < 0.05) increased in high RFI cows at mid and late lactation (Fig. 2b). A decreased (*P* < 0.05) concentration of methylhistidine was observed in high RFI cows at mid and late lactation stages. The concentration of PC aa C40:6 was significantly (*P* < 0.05) increased in high RFI cows at early and mid lactation. However, at late lactation, it tended to increase in high RFI cows.

### Multivariate analyses at each lactation stages

**Fig. 3:**
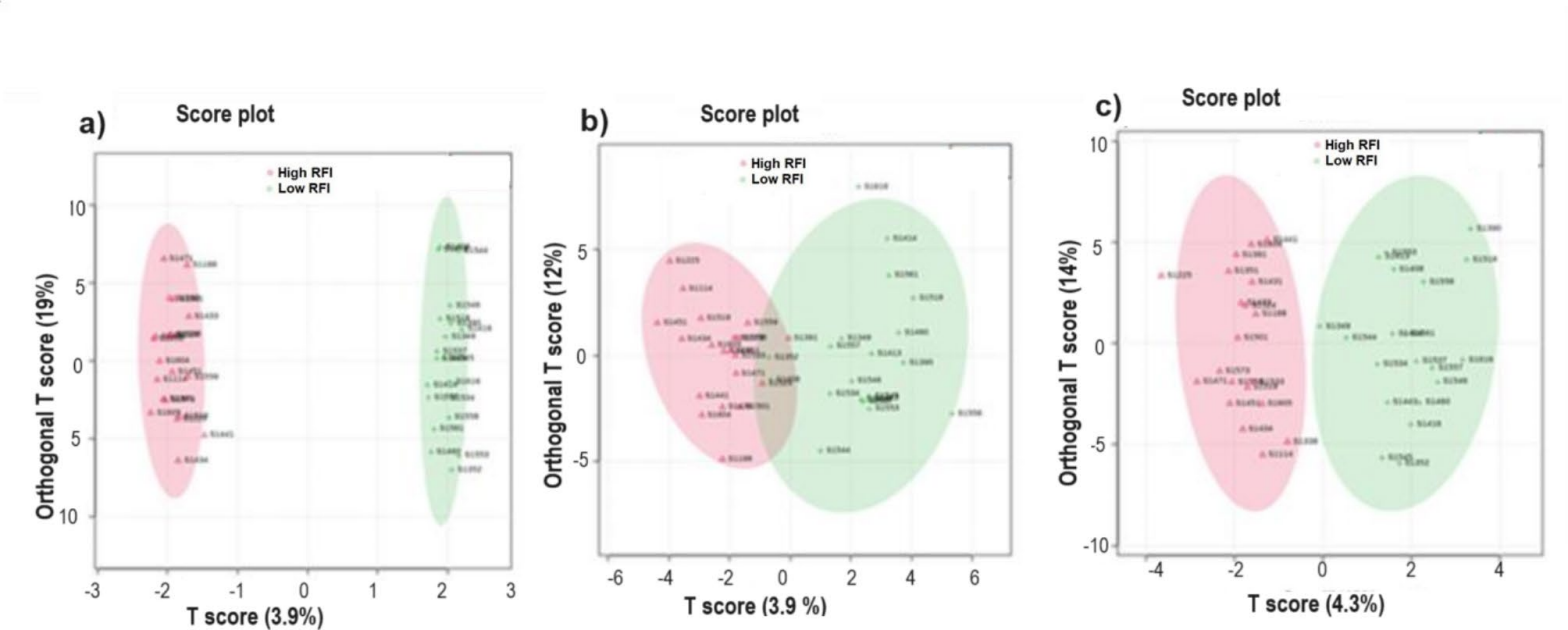
OPLS-DA score plot showing the grouping of the high (*n* = 20) and low RFI (*n* = 20) cows based on serum metabolite profiles at early (50.6 ± 4.6 DIM), mid (150.8 ± 2.6 DIM) and late (240.4 ± 3.8 DIM) lactation stages. The high RFI groups are shown by red colors and the low RFI groups are shown by green colors at early (**a**), mid (**b**) and late (**c**) lactation stages.

The orthogonal partial least squares discriminant analysis (OPLS-DA), which produced score plots showing the separation of the high and low RFI cows at three time points during the lactation period is shown in Fig. 3. At early and late lactation, the two RFI groups showed a clear separation based on the serum metabolite profiles (Fig. 3a and c), however, at mid lactation the high and low RFI cows formed two groups but they are not clearly separated (Fig. 3b). To confirm if the separations between the two groups were not due to chance, we performed permutation analyses and observed significant predictive relevance of the OPLS-DA models at early (Q^2^ = 0.40, *P* < 5e-04), mid (Q^2^ = 0.42, *P* < 5e-04) and late (Q^2^ = 0.21, *P* = 0.019) lactation stages.

**Fig. 4:**
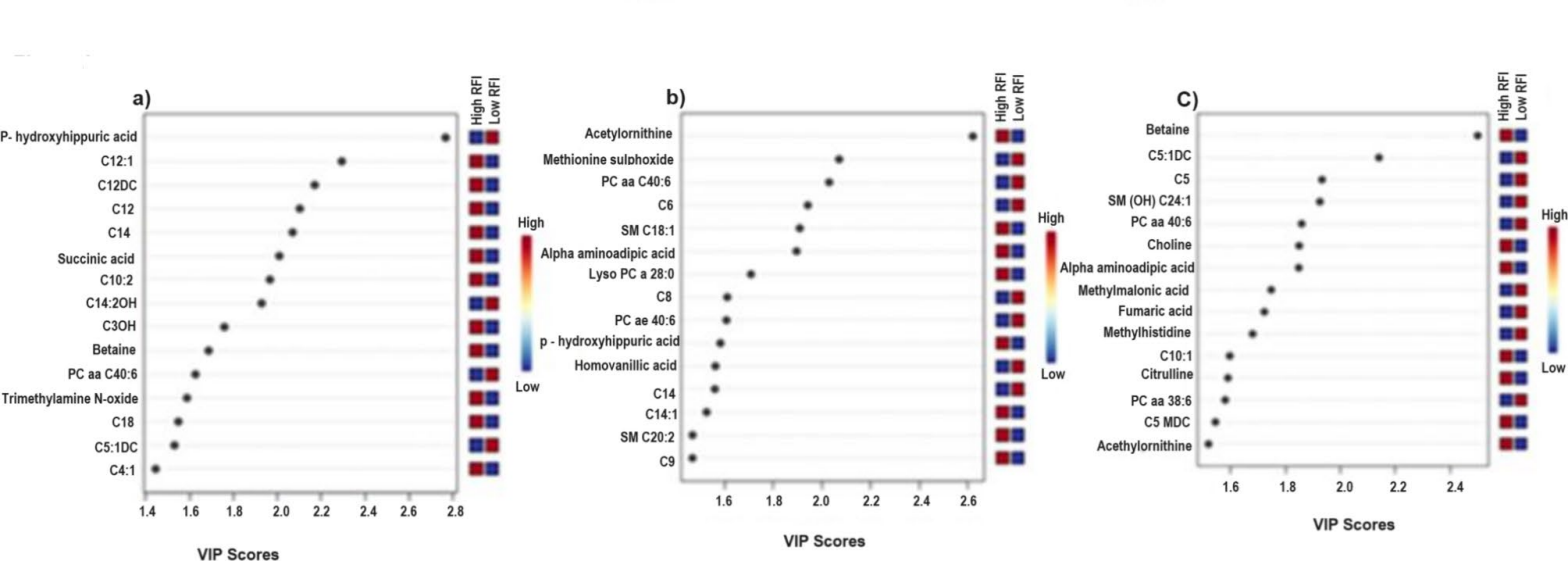
The top 15 metabolites responsible for the separation of high (*n* = 20) and low (*n* = 20) RFI groups at early (50.6 ± 4.6 DIM), mid (150.8 ± 2.6 DIM), and late (240.4 ± 3.8 DIM) lactation stages. The top 15 metabolites were ranked by VIP score (VIP > 1) in the discriminant analysis model. The serum concentration of each metabolite is shown by a heat map (blue = low, red = high concentration) on the right in high (*n* = 20) and low RFI (*n* = 20) Holstein cows at early (**a**), mid (**b**) and late (**c**) lactation stages.

The variable importance in projection (VIP) analyses shows the top 15 metabolites that were responsible for the separation between high and low RFI groups at early, mid, and late lactation (Fig. 4). At the early lactation stage, p-Hydroxyhippuric acid and medium and long chain acylcarnitines (C12:1, C12DC, C12 and C14) were the top 5 metabolites with VIP score > 2 (Fig. 4a). At mid lactation, acetylornithine, methionine-sulfoxide, PC aa C40:6, C6 and SM C18:1 were the top metabolites that distinguished the RFI groups with VIP > 2 (Fig. 4b). Betaine, C5:1-DC, C5, SM(OH) C24:1 and PC aa C40:6 were the top 5 metabolites that separated the RFI groups in late lactation with VIP score > 1.8 (Fig. 4c).

### Candidate biomarkers of RFI

Receiver operating characteristic (ROC) curve analysis revealed candidate biomarkers that distinguish high and low RFI groups at the early, mid, and late lactation stages (Table 4). P-Hydroxyhippuric acid (area under the curve (AUC) = 0.82), C12:1 (AUC = 0.79) and C14 (AUC = 0.77) were potential candidate biomarkers of RFI at the early lactation stage. At the mid lactation stage, acetylornithine was an excellent candidate biomarker with AUC of 0.93. SM C18:1 and lyso PC a C28:0 were also among the top 3 candidate biomarkers with AUC values of 0.85 and 0.78, respectively. Acetylornithine continued as the top candidate biomarker at the late lactation stage with decreased AUC value of 0.76 as compared to the mid lactation stage. Moreover, betaine and fumaric acid distinguished high and low RFI cows with similar AUC value of 0.76.

**Table 4:** The top 3 serum candidate biomarkers that distinguished high and low RFI cows at early (50.6 ± 4.6), mid (150.8 ± 2.6) and late (240.4 ± 3.8) lactation stages of Holstein cows.

Lactation stage	Metabolites	AUC	Confidence interval
Early lactation	p-Hydroxyhippuric acid	0.82	0.72–0.97
	C12:1	0.79	0.65–0.93
	C14	0.77	0.61–0.90
Mid-lactation	Acetylornithine	0.93	0.84–0.99
	SM 18:1	0.85	0.72–0.95
	lysoPC a C28:0	0.78	0.62–0.92
Late lactation	Acetylornithine	0.76	0.61–0.90
	Betaine	0.76	0.61–0.90
	Fumaric acid	0.76	0.60–0.89

### Predicting RFI phenotypes from serum metabolites

**Fig. 5:**
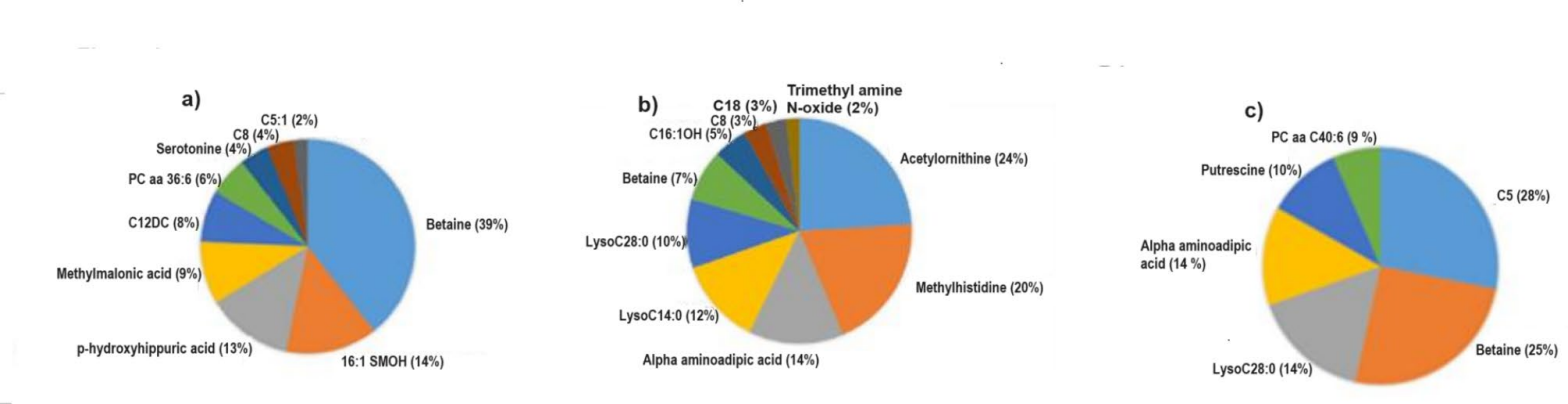
The relative contribution of explanatory variables in the RFI prediction model developed at early (50.6 ± 4.6), mid (150.8 ± 2.6) and late (240.4 ± 3.8) lactation stages. The proportion of variation in RFI explained by each metabolite in the model are shown in a pie charts at early (**a**), mid (**b**) and late (**c**) lactation stages of Holstein cows (*n* = 40).

A panel of 9 serum metabolites (betaine, SM (OH) C16:1, p-Hydroxyhippuric acid, methylmalonic acid, C12-DC, PC aa C36, serotonin, C8, C5:1) predicted RFI with R^2^ = 0.78 and validation R^2^^2^ (R^2^val) = 0.54, root mean squares of errors (RMSE) = 1.92 at early lactation stage. Betaine and SM (OH) C16:1 had a relative contribution of 39% and 11% to the RFI prediction model, respectively (Fig. 5a). At the mid lactation stage, a set of 10 metabolites (acetyl-ornithine, methylhistidine, alpha-aminoadipic acid, lysoPC a C14:0, lysoPC a C28:0, betaine, C16:1-OH, C8, C18 and Trimethylamine N-oxide) predicted RFI with model R^2^ = 0.76 and R^2^val = 0.68, RMSE = 1.78. Acetylornithine and methylhistidine had 25% and 20% relative contribution to the RFI prediction model, respectively (Fig. 5b). Similarly, at the late lactation stage, 6 metabolites (C5, betaine, lysoPC a C:28:0, alpha-aminoadipic acid, putrescine, PC aa C40:6) predicted RFI with R^2^ = 0.74 and R^2^val = 0.64, RMSE = 1.65. Interestingly, C5 and betaine were the most important metabolites in the RFI prediction model with 28% and 25% contributions, respectively (Fig. 5c). Betaine was the only metabolite that was consistently included in the prediction models accounting for 39, 7 and 25% of phenotypic variation in RFI across lactation stages.

## Discussion

This study was part of a large research project in which we reported the potential of milk metabolome to understand the physiology and identify biomarkers of RFI during the lactation period of Holstein cows^14^. Complementary to this, the current study utilized circulating serum samples to monitor metabolic differences, identify physiological biomarkers, and use them as predictors of RFI phenotypes in a cross-sectional setup during the lactation period. The comparison of the serum metabolite profiles between high and low RFI cows at early, mid, and late lactation stages revealed lactation stage-dependent physiological adaptations of more efficient cows to maintain similar production levels with less efficient cows from less feed.

Multivariate data analyses on longitudinal serum metabolome data revealed a clear separation between serum samples collected at early mid and late lactation stages. This has been shown by both unsupervised (PCA) and supervised (PLS-DA) analyses and agrees with the results of the PCA analysis undertaken in same lactation stages using milk samples^14^. Connolly et al.^15^ demonstrated significant differences in LC_MS/MS and^1^H - NMR based milk metabolite profiles across the stages of lactation, with early-lactation milk having a distinct metabolomics profile. A total of 269 and 17 metabolites had significantly different levels across lactation in LC_MS/MS and^1^H – NMR based metabolomics. In our study, the candidate biomarkers identified varied with lactation stage likely due to the difference in the regulation of nutrient partition in each stage of lactation. Interestingly, the low RFI group remained –RFI, and the high RFI group remained + RFI at all the three stages of lactation (Table 5) indicating that the possibility of re-ranking was minimal.

This result is consistent with the previous study in that we reported increased concentrations of medium and long-chain acyl-carnitines (C10:2, C10:1, C10, and C16:1-OH) in milk samples from high RFI cows (compared to the low RFI) at early lactation, however, the metabolite species were different^14^. In the current study, the early lactation stage was characterized by increased serum concentration of medium and long-chain acylcarnitines (C12, C12:1, C12DC, and C14) in high RFI cows. A study by Zhao et al.^16^ showed that the serum metabolome of the high fat mobilizing cows was characterized by higher concentrations of acylcarnitines. Huber et al.^17^ suggested that metabolic dysregulation and energetic imbalance may not be based on the detrimental effects of excessive lipid mobilization but on the consequences of less effective mitochondrial function. Kennedy et al.^18^ demonstrated that the high efficiency dairy cows had lower fatty acid oxidation and fatty acid oxidation per kilogram of body weight ^0.75^ compared to low efficiency during the AD-LIB period. Furthermore, the relative mitochondrial DNA copy numbers in the liver were lower in the low efficiency than the high efficiency group^18^. It should be noted that in our study, body fat mobilization parameters (e.g., serum NEFA) were not included and it is not possible to confirm if the high and the low RFI cows differ in fat mobilization during early lactation.

Differences in potentially preformed fatty acids (i.e., mobilized) should typically show an opposite trend compared to fatty acids synthesized de novo, if cows were mobilizing. We observed increased concentration of shorter acylcarnitines in low RFI group (compared high RFI) at mid lactation stage. Interestingly, serum levels of C:18 was significantly higher in low RFI cows (at mid lactation) suggesting a prolonged tissue mobilization that may have occurred in low RFI cows. The pattern of increased acylcarnitines in high RFI at early lactation and decreased concentrations in mid-lactation was also observed in the comparisons of milk acylcarnitines (high vs. low RFI) in milk samples^14^. Glycerophospholipids (SM C18:1, lysoPC a C28:1, SM C20:2, SM C16:1, SM C16:1-OH and SM C18:0) were increased in high RFI cows compared to the low RFI cows. Sphingomyelin has been shown to negatively influence lipid metabolism by affecting the binding or activity of lipoprotein lipase^19^, and it has been suggested that accelerated lipolysis was associated with sphengomyelin depletion^20^. The increased sphengomyelin in high RFI cows may be associated with relatively reduced (compared to low RFI) fat mobilization. This is consistent with the finding that estimated breeding values of residual energy efficiency and negative energy balance are positively correlated at mid-lactation^21^. Furthermore, ceramide and related products of sphingomyelin synthesis and breakdown are potent regulators of cell proliferation, activation, and apoptosis^22^. The increased serum concentration of sphingomyelin and its role in cellular proliferation and apoptosis suggests a higher metabolic rate in high RFI cows and agrees with the finding that 37% of the variation in RFI was explained by protein turnover^23^.

In the liver, lysophosphatidylcholines downregulate genes involved in hepatic fatty acid oxidation^24^. In our study, we observed increased concentrations of lysoPC a C28:0, lysoPC a C28:1, SM C18:1, and choline in high RFI cows suggesting that hepatic fatty acid oxidation may have been comparatively affected in high RFI cows. Interestingly, the serum concentration of fumaric acid was decreased in high RFI cows suggesting a lower rate of energy production given the role of fumaric acid in the TCA cycle as intermediates in the intracellular energy production in the form of ATP^25^. Muscle fatty acid oxidation declines with lactation^26^, and this decline may be higher in high RFI cows compared to the low RFI counterparts. The high RFI cows had higher concentrations of choline and betaine. Choline is biosynthesized in the liver via the phosphatidylethanolamine N-methyltransferase pathway^27^, and betaine is an oxidative intermediate of choline that supplies a methyl group for the conversion of homocysteine to methionine^28^. This suggests that the concentration of serum choline depends on its rate of biosynthesis and oxidation. The rate of choline biosynthesis might be higher than the rate of oxidation in high RFI cows, or there may be a lower rate of choline oxidation in high RFI cows as compared to the low RFI groups.

High RFI cows had a consistently increased serum concentration of betaine at early, mid, and late lactation stages. This result is in agreement with our previous report that showed an increased concentration of betaine in milk samples during the lactation period^14^. In addition, betaine is one of the metabolites that explains the variation in RFI at all three stages of lactation. However, the decline in betaine’s relative contribution to the variation in RFI at mid lactation could be explained by the physiological distinctness of the lactation stages that may allowed other metabolites to emerge relatively more important. In another study, Karisa and colleagues^11^ reported the association of betaine and RFI in beef cattle. A study in pigs also showed that piglets from two lines differed in their plasma concentrations of betaine which were greater in high RFI piglets than in low RFI piglets^29^. This may be explained by the increased oxidation of choline to betaine in high RFI cows compared to their low RFI counterparts. It is also likely that the low RFI cows may have utilized betaine at a higher rate than the high RFI cows, which subsequently led to comparatively decreased serum concentration in low RFI cows. The comparison of serum concentration of methylhistidine between the high and low RFI groups also showed an increased concentration in low RFI cows at the mid and late lactation stages, however, there was no difference at early lactation. The compound 3-methylhistidine is used as a marker of myofibrillar muscle protein breakdown because it is released into the circulation during muscle degradation and cannot be further metabolized or used for protein re-synthesis, but instead is excreted in the urine^30^. The results of this study showed an increasing trend in methylhistidine concentration during lactation (the lowest in early and the highest in late lactation) and this challenges the concept that muscle breakdown in dairy cows is restricted to the transition period with repletion occurring by wk 5 postpartum^31^. Komaragiri et al.^32^ reported a maximum amount of body tissue loss occurred between–2 and 5 wk postpartum and between 5 and 12 weeks postpartum, only small changes in body protein and fat were observed. Muscle breakdown seems to occur in dairy cows before parturition even when there isn’t negative energy balance^31^. Herd and Arthur^33^ reported that protein turnover and tissue metabolism and stress account for 37% of the variation in RFI in Angus steers. Sadri et al.^31^ argued that amino acids derived from muscle breakdown seem to be prioritized for anabolism to make the greater amount of amino acids for milk synthesis. This result disagrees with our previous observation that showed increased milk methylhistidine in high RFI cows at the late lactation stage^14^. The difference could be because the serum concentration of methyl histidine is a reflection of the entire body’s skeletal muscle breakdown^31^ while the milk methylhistidine concentration may predominantly indicate the mammary gland regression during late lactation. Moreover, the total mass of body muscle increases as lactation progresses, and the increase in serum methylhistidine in our study may reflect muscle turnover associated with greater muscle mass.

In this study, we observed a high similarity between the results of the multiple linear regression analyses and multivariate analyses. The set of metabolites that distinguished high and low RFI groups in the VIP analyses were among the metabolites that were significantly different between RFI groups in the regression analyses. Similarly, the candidate biomarkers identified using ROC curve analyses were also significantly different in the comparison of RFI groups using regression analyses. Acetylornithine emerged as the most relevant and discriminatory metabolite reflecting a potential role in feed efficiency with merit to be explored as a promising biomarker. We observed an increase in serum acetylornithine levels in high RFI cows at mid and late lactation. In agreement with this, Ghaffari et al.^30^ suggested that the hydrolysis of acetylornithine and subsequent production of ornithine could be influenced by the metabolic status of the cow.

Moderate accuracy of RFI prediction models was obtained for early (R^2^ = 0.78) mid (R^2^ = 0.76) and late (R^2^ = 0.74) lactation stages. The predictive performance of these models was much better than the previously reported predictive models. Poor predictive performance of RFI prediction models (R^2^ = 0.00 to 0.13) was reported by Martin et al.^13^ when descriptive, performance, sensor-derived behavior, and some blood analytes (glucose, BHB, triglycerides, and non-esterified fatty acids) were used as explanatory variables. Prediction models of RFI from milk spectra had low accuracy^34^. The improved performance for the prediction of RFI observed in our study might correlate with the role of predictor variables in the physiology of RFI. This further substantiates the suggestion that individual animal variation in RFI could be attributed to post-absorptive nutrient use efficiency^13^. In addition, comparable prediction accuracy of the models was observed in our previous study in milk samples^14^. In general, the findings of this study can be utilized in herd management or large-scale phenotyping of RFI. It should be noted that the prediction models were developed using the same animals (*n* = 40) used in the first analysis to identify significantly different metabolites between RFI groups. This may have resulted in a less conservative prediction accuracy of the models, even if, LOOCV was used to estimate the performance of the prediction models on data that was not used to train the models. Therefore, validation of the results in independent samples could reaffirm the biomarkers and prediction models for practical utility.

In conclusion, the study showed that metabolic adaptation for nutrient efficiency is variable during lactation. Fat and other tissue mobilization is apparent during early lactation, and feed-efficient cows appear to have a better rate of fat oxidation and TCA cycle evidenced by lower concentrations of medium and long chain acylcarnitines. In mid lactation stage, however, more efficient cows had higher concentrations of markers of fat oxidation and may have mobilized much higher fat than the less efficient cows. The mechanism of metabolic adaptation at late lactation is likely a higher muscle protein breakdown in more efficient cows, and this has been shown by comparatively higher concentration of methylhistidine (a marker of muscle break down). However, in this study, energy balance and body reserve mobilization data were not included and this has limited the interpretation of the results. The serum concentration of betaine was consistently increased in high RFI cows at all three-time points during the lactation. We further explored the potential of circulating serum metabolites as biomarkers and predictors of RFI. Overall, the study highlighted the physiological mechanisms of RFI, candidate biomarkers and prediction models based on serum metabolome profile. These promising results, however, need to be validated in an independent set of lactating dairy cows.

## Materials and methods

### Animals and diet

The study was undertaken in 72 mixed parity lactating Holstein cows (29 primiparous and 43 multiparous) at the University of Alberta, Dairy Research and Technology Center (DRTC) from June 2017 to October 2018. All cows in the experiment were individually housed in a ventilated tie-stall barn with access to water. Cows were fed a total mixed ration (TMR) and a daily ration was offered for ad libitum intake to allow approximately 5% feed refusals throughout the experiment. All cows were fed once daily in the morning at 0800 h. Individual offered feed weight in the morning and refusal feed weight left on the next morning were recorded daily. From all 72 cows, daily feed intake, daily milk yield, monthly body weight, and weekly milk samples were collected from 3 to 240 days in milk (DIM). The milk samples were collected to obtain fat, protein, and lactose concentrations that were used in the milk production energy requirement calculation. In addition, parity (P), age at first calving (AFC), health status (HS), month and year of sampling (MY) were recorded. Cows that were culled/died due to disease/s or any other reason before 240 DIM were excluded from the experiment. Disease incidence in the window of 2 weeks before and after each sampling dates was considered in the analysis. The details of data collection, diet composition, and ingredients, and disease occurrence are described in our previous study^14^. All the experimental procedures for this study were approved by the University of Alberta Animal Policy and Welfare Committee for Livestock (AUP00000170), and animals were cared for in accordance with the guidelines of the Canadian Council on Animal Care^35^. All methods were performed in accordance with the relevant guidelines and regulations. In addition, the study is reported in accordance with ARRIVE guidelines.

### Data collection and processing for RFI estimation

The parameters that are required to calculate RFI include; daily actual energy intake (AEI), weekly milk production energy requirement (MPER), metabolic body weight (MBW), and empty body weight change (EBWC). These parameters were derived from recorded raw data for each animal in the study. Actual energy intake was derived from the daily DMI recorded (3-240 DIM) as a product of the daily DMI and net energy density of the diet. The daily DMI was obtained as the product of the daily feed intake by dry matter percentage of the diet. Metabolic body weight was calculated as BW powered to 0.75^36^. Empty body weight was an adjusted BW for the gut fill (GF), and it is the function of individual daily DMI and the metabolizable energy content of the diet that each animal consumed at the test day^36^. It is calculated as EBW (kg) = BW − GF, where GF (kg) = DMI × [11 − (7 × MED/15)], in which MED was the metabolizable energy density (Mcal∙kg − 1) of the diet^37^ (NRC 2001). Empty body weight was used to calculate empty body weight change. Milk production energy requirement is the sum of the heat of combustion of milk fat, protein, and lactose and calculated as MPER (Mcal∙d − 1) = {[0.0929 × fat (%)] + [0.0547 × CP (%)] + [0.0395 × lactose (%)]} × milk yield^37^. To calculate daily RFI from 3 to 240 DIM, the calculated weekly MPER and monthly EBW were used to predict daily values. The details of the derivation of parameters for RFI calculation, prediction of daily values for MPER and EBW using a random regression model were described in our previous study^38^. After predicting daily values of EBW from monthly values, empty body weight change (EBWC) was calculated as a difference in EBW between two consecutive days (3-240 DIM). The details are described in our previous study^39^.

### RFI calculation and identifying RFI groups

Residual feed intake was calculated in 72 lactating Holstein dairy cows according to our previous study^39^. In short, RFI values were calculated as the difference between the actual (AEI) and expected net energy intake (EEI). Phenotyping for RFI required recording of daily DMI and predicting EEI accounting for multifunctional energy requirements (metabolic body weight (MBW), empty body weight (EBW), empty body weight change (EBC) and milk production net energy requirements (MPER)). Mixed parity cows were used in the study and parity (P) was included in the RFI calculation model. A multiple linear and quadratic regression model was used to predict EEI values from 3 to 240 DIM. The smoothed total AEI was linearly regressed on a total of 237 days predicted traits of MBW, MPER, and EBWC and parity to obtain the individual’s 237 days of EEI and RFI. The daily average lactation RFI for each individual over 237 days was obtained by dividing the total RFI by animal days in the record. Then, the 72 RFI predicted cows were ranked and categorized into low RFI (RFI < 0.5 SD from the mean, *n* = 21), mid RFI (*n* = 27) and high RFI (RFI > 0.5 SD from the mean = 24). Mid RFI samples were removed from all analyses and all comparisons were made between low (*n* = 20) and high RFI (*n* = 20) groups. One cow from low RFI and 4 cows from the high RFI groups were excluded to maintain a balanced sample size between the comparison groups. Furthermore, we calculated weekly RFI (averages of 7 days of RFI from weeks 1–34) for each cow. The weekly average RFI for high and low RFI groups at early, mid and late lactation stages are described in Table 5. In addition, fat (%), protein (%), total solids (%), milk BHB (mmol/L), acetone (mmol/L) and somatic cell score (10^3^/L) were analyzed every week. For this study, milk component analysis results in the week of blood sampling were used. Milk production (kg) on the blood sampling days were used to calculate average values for the RFI groups.

**Table 5:** Summaries (weekly averages and SD) of production and feed efficiency parameters recorded at early (50.6 ± 4.6 DIM), mid (150.8 ± 2.6 DIM) and late (240.4 ± 3.8 DIM) lactation stages of Holstein cows were presented in high (*n* = 20) and low (*n* = 20) RFI groups.

Parameters	Early lactation		Mid lactation		Late lactation	
	High RFI	Low RFI	High RFI	Low RFI	High RFI	Low RFI
Residual feed intake (kg DMI/d)	1.23 ± 1.8	-1.48 ± 2.28	1.75 ± 1.83	-2.2 ± 3.48	3.04 ± 3.60	-0.91 ± 3.2
Milk production (kg)	41 ± 10.8	40.8 ± 9.6	39.4 ± 10.1	37.0 ± 6.7	37.1 ± 6.2	36 ± 8.7
Milk fat (%)	3.73 ± 0.47	3.69 ± 0.35	3.92 ± 0.76	3.86 ± 0.6	4.08 ± 0.67	3.95 ± 1.16
Milk protein (%)	3.0 ± 0.16	2.88 ± 0.25	3.41 ± 0.26	3.21 ± 0.2	3.45 ± 0.25	3.35 ± 0.28
Lactose (%)	4.66 ± 0.13	4.65 ± 0.15	4.57 ± 0.11	4.62 ± 0.1	4.57 ± 0.15	4.55 ± 0.16
Milk BHB (mmol/L)	0.08 ± 0.04	0.08 ± 0.03	0.09 ± 0.03	0.09 ± 0.1	0.08 ± 0.03	0.09 ± 0.04
Somatic cell score (10^3^/L)	39.6 ± 44.5	52.23 ± 69.5	66.6 ± 61.6	72 ± 73.8	117 ± 206	142 ± 217
Total solids (%)	12.4 ± 0.57	12.22 ± 0.68	12.7 ± 0.85	12.9 ± 0.7	13.1 ± 0.77	12.9 ± 1.22
Acetone (mmol/L)	0.14 ± 0.05	0.14 ± 0.04	0.14 ± 0.05	0.2 ± 0.06	0.12 ± 0.04	0.14 ± 0.04

### Serum sample collection for LC-MS/MS analyses

Blood samples were collected from the coccygeal veins of 72 lactating dairy cows at early (50.6 ± 4.6 DIM), mid (150.8 ± 2.6 DIM), and late (240.4 ± 3.8 DIM) lactation stages. Mixed parity cows with parities ranging from 1 to 5 were used in the study. Blood samples were collected from all the 72 cows after morning milking and before feeding (6:00 h). All blood samples were collected into 10 mL vacutainer tubes (Becton Dickinson, Franklin Lakes, NJ, USA) and allowed to coagulate at room temperature. Immediately after coagulation, the tubes were centrifuged at 2090 g at 4 °C for 20 min to separate the serum (Rotanta 460 R centrifuge, Hettich Zentrifugan, Tuttlingen, Germany). Subsequently, the separated serum was aspirated from the supernatant gradually by transfer pipets (Fisher Scientific, Toronto, ON, Canada) into a sterile 2 mL tube (Fisher Scientific, Toronto, ON, Canada). Serum samples were stored in a − 80 °C freezer until DI/LC-MS/MS analyses.

### TMIC Prime Assay DI/LC-MS/MS method

We applied a targeted quantitative metabolomics approach to analyze the serum samples using a combination of direct injection mass spectrometry with a reverse-phase LC-MS/MS custom assay. This custom assay (The Metabolomics Innovation Centre (TMIC), University of Alberta), in combination with an ABI 4000 Q-Trap (Applied Biosystems/MDS Sciex) mass spectrometer, can be used for the targeted identification and quantification of endogenous metabolites including amino acids, acylcarnitines, organic acids, biogenic amines & derivatives, uremic toxins, glycerophospholipids, sphingolipids and sugars. The method combines the derivatization and extraction of analytes, and the selective mass-spectrometric detection using multiple reaction monitoring (MRM) pairs. Isotope-labeled internal standards and other internal standards were used for metabolite quantification. The custom assay contains a 96 deep-well plate with a filter plate attached with sealing tape, and reagents and solvents used to prepare the plate assay. The first 14 wells were used for one blank, three zero samples, seven standards, and three quality control samples. For all metabolites except organic acid, serum samples were thawed on ice and were vortexed and centrifuged at 13,000x g. 10 µL of each sample was loaded onto the center of the filter on the upper 96-well plate and dried in a stream of nitrogen. Subsequently, phenyl-isothiocyanate was added for derivatization. After incubation, the filter spots were dried again using an evaporator. Extraction of the metabolites was then achieved by adding 300 µL of extraction solvent. The extracts were obtained by centrifugation into the lower 96-deep well plate, followed by a dilution step with MS running solvent.

For organic acid analysis, 150 uL of ice-cold methanol and 10 uL of isotope-labeled internal standard mixture was added to 50 uL of serum sample for overnight protein precipitation. Then it was centrifuged at 13000x g for 20 min. 50 uL of supernatant was loaded into the center of wells of a 96-deep well plate, followed by the addition of 3-nitrophenylhydrazine (NPH) reagent. After incubation for 2 h, BHT stabilizer and water were added before LC-MS injection.

Mass spectrometric analysis was performed at TMIC on an API4000 Qtrap^®^ tandem mass spectrometry instrument (Applied Biosystems/MDS Analytical Technologies, Foster City, CA) equipped with an Agilent 1100 series HPLC system (Agilent Technologies, Palo Alto, CA). The samples were delivered to the mass spectrometer by an LC method followed by a direct injection (DI) method. Data analysis was done using Analyst 1.6.2.

### Statistical data analyses

Serum samples from high RFI (*n* = 20) and low RFI (*n* = 20) lactating dairy cows were examined at early, mid, and late lactation stages. We employed a targeted quantitative metabolomics approach (direct injection mass spectrometry with a reverse-phase LC-MS/MS custom assay), and quantified 100 serum metabolites at the three time points during lactation. We performed multiple linear regression analyses to quantitatively compare the metabolite profiles between high and low RFI groups while taking into account the fixed effects. The model was fitted for each serum metabolite concentration as dependent variable and RFI groups, parity, age at first calving, health status, month and year of sampling time as independent variables using R packages. The metabolites were analyzed using the following model:

$${\text{Yijklmn}} = \upmu + {\text{Ri}} + {\text{Pj}} + {\text{MYk}} + {\text{AFCl}} + {\text{HSm}} + {\text{eijklmn}},$$

where Yijklmn is the nth observation (individual serum concentration) for the cow tested from the ith RFI group (low and high RFI) and jth parity (1–3+), MYk is the effect of the kth Month Year combination, AFCl is the effect of lth age at first calving, HSm is the effect of mth health status and eijklm is the deviation for the ijklmth observation or error term. In the analyses, all cows with parity > 3 were categorized as ‘3+”. Each metabolite was tested for normal distribution and where appropriate, for data that were not normally distributed, the natural log-transformation was used and back-transformed results were presented. The results were presented as least squares means ± standard error of mean per RFI category. Significance was declared at P < 0.05 and tendencies were declared at 0.05 ≤ P < 0.1.

Serum metabolites that were significantly different in the comparisons between high and low RFI groups at early, mid, and late lactation stages were used to predict RFI value. Average RFI values were used as the dependent variable and significantly different metabolites as independent variables in the multiple regression analyses. Before the regression analyses, the serum metabolite concentrations were adjusted for the significant fixed effects that include parity, sampling month and year, health status, and age at first calving. Leave-one-out cross-validation (LOOCV) was done using the Caret package in R^40^ to assess the models’ predictive quality.

Multivariate analyses were performed using the Metaboanalyst web-based platform^41^. Orthogonal partial least squares discriminant analysis (OPLS-DA) was used to test whether the high and low RFI groups cluster separately based on serum metabolite profiles. To minimize the possibility that the observed separation for an OPLS-DA plot was due to chance, we performed permutation testing that involved repeated (2,000 times) OPLS-DA calculations using different random labeling of the samples. A significant P-value (< 0.05) indicates that the separation observed between the two groups is unlikely due to chance. Receiver-operator characteristic curves (ROC) are often summarized into a metric called the area under the curve (AUC). The AUC can be interpreted as the probability that a diagnostic test or a classifier will rank a randomly chosen positive instance higher than a randomly chosen negative one. For this study, we presented the top three metabolites (based on AUC values) at the early, mid and late lactation stages. In addition, principal component (PCA), partial least squares discriminant analysis (PLS-DA) and VIP were undertaken on longitudinal data to identify if the early, mid and late lactation stages cluster separately based on the serum metabolite profiles. The VIP analysis was used to identify the top metabolites that significantly distinguish the three lactation stages.

## Data Availability

The data that support the findings of this study are not openly available and are available from the corresponding author upon reasonable request.
